# Oncogenic Orphan Nuclear Receptor NR4A3 Interacts and Cooperates with MYB in Acinic Cell Carcinoma

**DOI:** 10.3390/cancers12092433

**Published:** 2020-08-27

**Authors:** David Y. Lee, Kathryn J. Brayer, Yoshitsugu Mitani, Eric A. Burns, Pulivarthi H. Rao, Diana Bell, Michelle D. Williams, Renata Ferrarotto, Kristen B. Pytynia, Adel K. El-Naggar, Scott A. Ness

**Affiliations:** 1 Department of Internal Medicine, Division of Hematology/Oncology, Section of Radiation Oncology, University of New Mexico School of Medicine, Albuquerque, NM 87131, USA; dylee@salud.unm.edu (D.Y.L.); ErBurns@salud.unm.edu (E.A.B.); 2 Department of Internal Medicine, Division of Molecular Medicine, University of New Mexico School of Medicine, Albuquerque, NM 87131, USA; KBrayer@salud.unm.edu; 3 Department of Pathology, University of Texas MD Anderson Cancer Center, Houston, TX 77030, USA; yomitani@mdanderson.org (Y.M.); diana.bell@mdanderson.org (D.B.); mdwillia@mdanderson.org (M.D.W.); 4 Department of Pediatrics, Texas Children’s Cancer and Hematology Center, Texas Children’s Hospital, Baylor College of Medicine, Houston, TX 77030, USA; PHRAO@texaschildrens.org; 5 Department of Thoracic and Head and Neck Medical Oncology, University of Texas MD Anderson Cancer Center, Houston, TX 77030, USA; rferrarotto@mdanderson.org; 6 Department of Head and Neck Surgery, University of Texas MD Anderson Cancer Center, Houston, TX 77030, USA; KBPytynia@mdanderson.org; 7 Comprehensive Cancer Center, University of New Mexico, Albuquerque, NM 87131, USA

**Keywords:** oncogene cooperativity, enhancer hijacking, protein interactions, oncogenesis, bioinformatics

## Abstract

Acinic cell carcinoma (AcCC) is a morphologically distinctive salivary gland malignancy often associated with chromosome rearrangements leading to overexpression of the NR4A3 transcription factor. However, little is known about how NR4A3 contributes to AcCC biology. Detailed RNA-sequencing of 21 archived AcCC samples revealed fusion reads arising from recurrent t(4;9), t(9;12), t(8;9) or t(2;4) chromosomal translocations, which positioned highly active enhancers adjacent to the promoter of the *NR4A3* gene or the closely related *NR4A2* gene, resulting in their aberrant overexpression. Transcriptome analyses revealed several distinct subgroups of AcCC tumors, including a subgroup that overexpressed both *NR4A3* and *MSANTD3*. A poor survival subset of the tumors with high-grade transformation expressed *NR4A3* and *POMC* as well as *MYB*, an oncogene that is the major driver in a different type of salivary gland tumor, adenoid cystic carcinoma. The combination of *NR4A3* and *MYB* showed cooperativity in regulating a distinct set of genes. In addition, the ligand binding domain of NR4A3 directly bound the Myb DNA binding domain. Transformation assays indicated that, while overexpressed NR4A3 was sufficient to generate transformed colonies, the combination of NR4A3 plus Myb was more potent, leading to anchorage-independent growth and increased cellular invasiveness. The results confirm that *NR4A3* and *NR4A2* are the main driver genes of AcCC and suggest that concurrent overexpression of *NR4A3* and *MYB* defines a subset of AcCC patients with high-grade transformation that display exceptionally poor outcome.

## 1. Introduction

Acinic cell carcinoma (AcCC) is the fourth most common subtype of salivary gland tumors in adults and the most common subtype in children and young adults [1,2]. It is a morphologically distinctive salivary malignancy: a salient and distinguishing feature is the presence of acinar cells with cytoplasmic granules mimicking those of serous salivary glands. However, AcCC displays widely varied structural forms including conventional acinar, microcytic, tubulopapillary and macrocytic forms [3,4], suggesting underlying biologic heterogeneity. As a relatively indolent neoplasm, AcCC usually has a good prognosis [5,6,7]. It is generally managed with surgical excision, and adjuvant radiation therapy is utilized in patients with positive margins, perineural invasion or lymph node involvement. However, a subgroup of patients displays much more aggressive features including lymph node involvement, metastasis or high-grade transformation, but the molecular mechanisms leading to more aggressive phenotypes are unknown, and no biomarkers are available to identify the subgroup of patients who could benefit from more intensive treatments.

Recently, genomics studies implicated recurrent t(4;9) and t(12;20) translocations leading to the formation of *HTN3-MSANTD3* and *PRB3-ZNF217* fusion genes in some AcCC tumors [8]. However, more extensive analyses revealed recurrent translocations that juxtapose enhancers next to the *NR4A3* gene, suggesting that overexpression of *NR4A3* is the most common oncogenic driver event in AcCC [9,10,11]. The *NR4A3* (*NOR1*) gene and the highly related *NR4A2* (*NURR1*) gene encode nuclear orphan receptors and are part of the Nur77 steroid–thyroid hormone–retinoid receptor superfamily. Like other nuclear receptors, they encode transcription factors with conserved DNA-binding domains and C-terminal ligand-binding regulatory domains, but natural ligands regulating the NR4Ax proteins have not been identified [12,13]. The conserved domains could also be important for interacting with other transcription factors or coregulators [14,15]. Translocations that fuse the *NR4A3* and *EWSR1* genes occur in a majority of extraskeletal myxoid chondrosarcomas, and the resulting *EWSR1-NR4A3* fusion protein is thought to be the driver oncogene for most such tumors [16,17,18].

The use of optimized RNA-sequencing (RNA-seq) methods has been shown to be a useful approach to identify fusion transcripts in salivary gland tumors and to derive valuable gene expression signatures for identifying subgroups of tumors with different molecular and clinical phenotypes, even when relatively old archived samples must be used to have sufficient follow-up information [19,20,21]. Here, we use RNA-seq to identify fusion transcripts and gene expression signatures for a cohort of AcCC tumor samples. The results provide evidence for recurrent translocations in most, if not all AcCC tumors that lead to the transcriptional activation of the *NR4A3* gene or the highly related *NR4A2* gene. The transcriptome analysis revealed several subgroups of AcCC tumors with different gene expression profiles, including a previously unknown subset of the tumors with high-grade transformation that displayed very poor prognosis. Those tumors express *NR4A3* and the oncogene *MYB* and several types of evidence suggest that the NR4A3 and Myb proteins interact and that the two transcription factors cooperate to regulate genes that contribute to poor prognosis. These findings strengthen the conclusion that *NR4A3* is the most common driver oncogene in AcCC tumors and provide novel targets for possible development of biologic and therapeutic strategies for the management of patients with AcCC.

## 2. Results

### 2.1. AcCC Tumors Harbor Recurrent Translocations Involving the NR4A3 and NR4A2 Genes

We used optimized RNA-seq methods [20] to analyze a cohort of 23 acinic cell carcinoma (AcCC) samples, which yielded high quality results for 21 samples. Table 1 summarizes the clinicopathologic characteristics of the final cohort, which included 4 males and 17 females who ranged in age from 14 to 88 years, with mean age of 52. Twenty tumors arose from parotid gland and one from maxillary gland. They ranged in size from 1 to 6.5 cm with a mean of 3.1 cm. Tumors manifested the classical phenotypic characteristics of AcCC with variable structural and cellular composition. Only 5 patients had recurrent disease or metastases after primary surgical resection. (Additional clinical information is provided in Table S1.)

**Figure 1:**
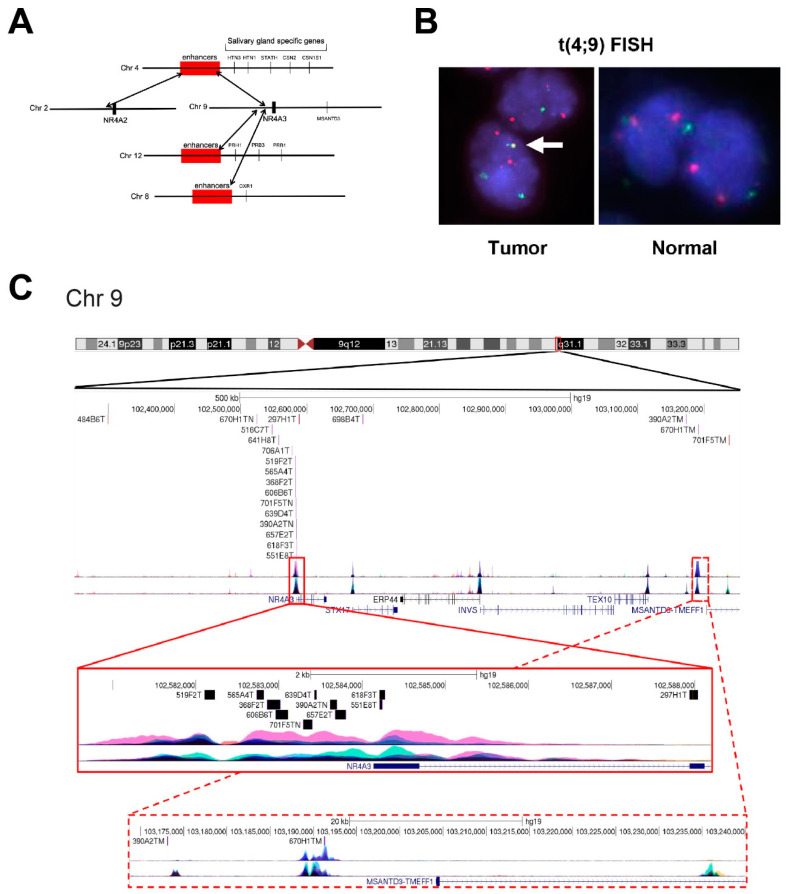
Chromosome fusions in AcCC tumors. (**A**) Diagram summarizes the fusion reads and putative translocations detected by RNA-seq analysis; (**B**) fluorescence in situ hybridization (FISH) analysis to verify the t(4;9) translocation was performed for chromosome 4 (green) and chromosome 9 (red). Yellow signal (arrow) in the tumor is the fusion chromosome; (**C**) chromosome 9 breakpoints. Diagram of the chromosome 9q31.1 region, with locations of translocations identified by RNA-seq fusion reads indicated. Note the clustering of breakpoints in the *NR4A3* gene promoter region. Two tumors also had fusion reads that mapped to the *MSANTD3* region at right. The insets at bottom show zoomed views of the *NR4A3* and *MSANTD3* gene regions and the breakpoints near each. Colored peaks indicate H3K27Ac (top) and H3K4Me3 (bottom) peaks from UCSC genome browser tracks (not determined in AcCC cells).

We first used the RNA-seq data to identify potential fusion reads that could indicate chromosomal translocations. We identified fusion reads that mapped upstream of the *NR4A3* gene on chromosome 9 in 16 of the 21 (76%) AcCC tumors (Table S2), all of which expressed high levels of *NR4A3*. As summarized in Figure 1A, the fusion reads identified a cluster of putative translocation breakpoints focused within a few kilobases upstream of the *NR4A3* gene. Interestingly, in one sample we instead identified fusion reads that mapped upstream of the related *NR4A2*-gene and that sample overexpressed *NR4A2* instead of *NR4A3*. Two of the AcCC samples that contained fusion reads mapping to the *NR4A3* gene also had some fusion reads that mapped to the nearby *MSANTD3* gene, and those samples overexpressed both *MSANTD3* and *NR4A3*. As shown in Figure 1A, *NR4A3* and *MSANTD3* are located in the same region of chromosome 9, suggesting that, at least in some AcCC samples, chromosomal translocations may lead to the aberrant activation of both genes.

Since *MSANTD3* has been described as a possible driver oncogene in AcCC, we downloaded the publicly available RNA-seq fastq and Chimeriscan results files for three AcCC samples previously analyzed by Barasch et al. [8]. Two of the three samples (SRR3056922 and SRR3056923) had been reported to have strong *MSANTD3* expression as detected by IHC, however only one (SRR3056922) was reported to have a t(4;9) translocation to the *MSANTD3* gene [8]. In our analysis of their data, both samples had fusion reads that mapped upstream of *NR4A3* (Table S3). Like the original authors, we found no evidence of the t(4;9) translocation in the third sample (SRR3059624), although it too had high levels of *NR4A3* expression. Thus, all AcCC samples analyzed had fusions involving the *NR4A3* or *NR4A2* genes.

### 2.2. Confirmation of t(4;9) Translocations by Fluorescence In Situ Hybridization

We performed fluorescence in situ hybridization (FISH) analysis to confirm the translocations identified by RNA-seq. As shown in Figure 1B, hybridization with Bacterial Artificial Chromosome (BAC) clones specific for chromosome 4 (green) and chromosome 9 (red) led to the detection of both normal chromosomes and fusion chromosomes (yellow, arrow) in AcCC samples. FISH analyses confirmed t(4;9) translocations in all tumors that were initially identified by RNA-seq (Table S2). One of the t(2;4) translocations were also verified by FISH (Table S2) using chromosome 2 and 4 BAC clones. Based on the extensive evidence from both RNA-seq and FISH, we conclude that AcCC tumors harbor recurrent translocations that activate the *NR4A3* or *NR4A2* genes, which are likely to be the driver oncogenes for these tumors. Our results, based only on RNA-seq, confirm the work published by others who used whole genome sequencing to identify t(4;9) translocations in AcCC tumors [9].

All of the fusion reads that we identified connected distant chromosomes to the upstream regions of the *NR4A3* or *NR4A2* genes. Our results agreed with the previous analysis suggesting that the *NR4A3* and *NR4A2* genes were activated through a mechanism of enhancer hijacking, in which the translocations juxtapose a highly active enhancer to the vicinity of the oncogene, leading to its overexpression [9]. This is summarized by Figure 1C, which shows that most of the translocation breakpoints cluster in a region just upstream of the *NR4A3* gene.

**Figure 2:**
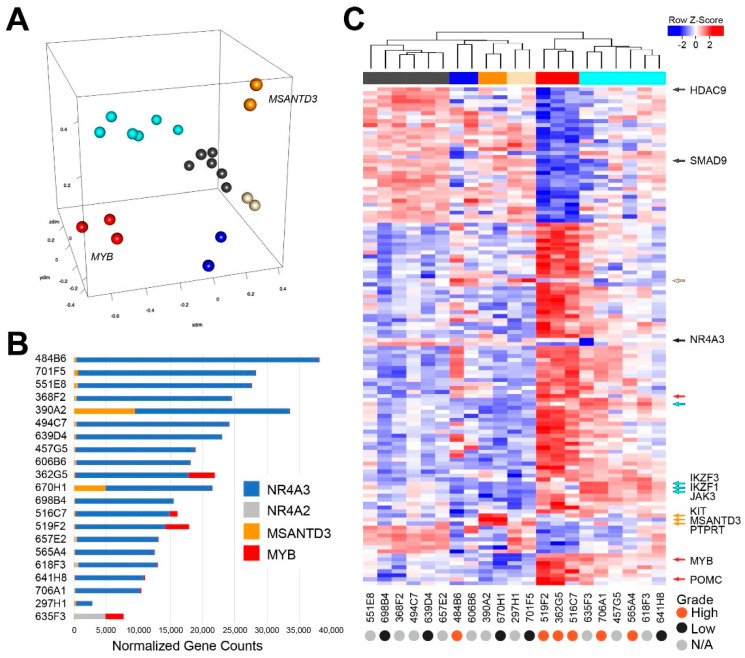
RNA sequencing analysis of AcCC tumors. (**A**) Multidimensional scaling identifies several distinct groups of AcCC samples (shaded with different colors); (**B**) bar chart summarizes the normalized expression levels of *NR4A3* (dark blue), *NR4A2* (gray), *MSANTD3* (orange) and *MYB* (red); (**C**) heatmap summarizes the most significant differences in gene expression among the six groups of AcCC tumors identified by hierarchical clustering, shown by the dendrogram and color bar at the top, which corresponds to the groups in panel A. Arrows along the right highlight genes characteristic for each group. Up- and downregulated genes are indicated by red and blue, respectively, as indicated in the key. Samples that had undergone high-grade transformation are indicated at bottom by orange dots. A full-sized heatmap with all the genes labeled is provided in Figure S2.

In contrast, the other ends of the fusion reads were spread out over much larger regions of several chromosomes. As shown in Figure S1 and summarized in Table 2, most of the fusion reads mapped to regions of chromosome 4 or 12 containing several genes that are very highly expressed in salivary gland tissue, including *HTN1*, *HTN3*, *STATH*, *CSN1S1* and *CSN2* on chromosome 4 or *PRH2*, *PRB1* and *PRB3* on chromosome 12. Altogether, we identified 12 AcCC samples with 6 different types of recurrent t(4;9) fusions, 4 AcCC samples with 3 different types of t(9;12) translocations, and one sample with a t(8;9) translocation, all of which appeared to activate the *NR4A3* gene. (See Figure 2, below) Our results confirm that AcCC tumors most often result from translocations that juxtapose enhancers to be near the *NR4A3* gene [9,10,11]. We also identified 1 sample with t(2;4) translocations linked to the *NR4A2* gene. Thus, translocations that activated the *NR4A3* or *NR4A2* genes were detected in 18 of the 21 samples. While the fusion reads we identified can be used to map putative translocation breakpoints, it is important to note that we did not find any evidence suggesting that novel *NR4A3* fusion proteins would be produced by the AcCC tumors, which contrasts with translocations in other tumor types that encode *NR4A3* fusion proteins [22]. Thus, the translocations appear to lead to overexpression of the otherwise normal *NR4A3* or *NR4A2* genes and should produce normal protein products.

### 2.3. Transcriptome Analysis Reveals Heterogeneity in Acinic Cell Carcinoma

Next we characterized the gene expression data for the AcCC samples. As shown in Figure 2A, multidimensional scaling separated the AcCC tumors into 6 distinct groups, suggesting underlying heterogeneity or the presence of subgroups of tumors with distinguishable gene expression profiles. The bar chart in Figure 2B shows that all of the AcCC samples expressed high levels of *NR4A3* (blue). The only exception was sample 653F3, which expressed *NR4A2* (gray) instead and is also the sample with the t(2:4) translocation involving *NR4A2* (Table S2). *MSANTD3* (orange) was expressed in two samples (390A2 and 670H1), which are also the two samples that had fusion reads linking the *MSANTD3* gene to the enhancer regions (Table S2). However, both samples that expressed *MSANTD3* expressed even higher levels of *NR4A3*.

We performed differential gene expression analysis to characterize the different groups of AcCC tumors. A heatmap showing some of the dramatic differences in gene expression between the groups is shown in Figure 2C (a larger version with all the genes labeled is in Figure S2 and complete gene expression information is provided in Table S4). Note: Although *NR4A3* is shown in the heatmap (arrow), its expression does not appear to be dramatically different across the AcCC samples, since it is highly expressed in all of them, except 653F3, which expresses *NR4A2* instead. Each subgroup of AcCC tumors identified in the 3D-plot in Figure 2A displayed a characteristic gene expression pattern in the heatmap. The group marked by the dark gray color bar in the heatmap and located at far left, displayed a unique gene expression profile and had several important upregulated genes, including *HDAC9* and *SMAD9* (arrows and see Figure S2). Elevated expression of *MSANTD3* was clearly distinguished in the two samples in the orange group, 390A2 and 670H1, which also overexpressed *KIT* and *PTPRT* (orange arrows and see Figure S2). The group at far right, indicated by the cyan color bar, were distinguished by overexpressed *IKZF1*, *IKZF3* and *JAK3* (cyan arrows and see Figure S2).

Another remarkable group, marked by the red color bar at the top of the heatmap and red dots at lower left in the 3D plot in Figure 2A, displayed high levels of *MYB* in addition to *NR4A3* and overexpressed the *POMC* gene, which is a known target gene for Nur-family transcription factors like NR4A3 [23,24,25]. As will be discussed in more detail below, the *MYB*-expressing (red) subgroup is a poor survival group of AcCC tumors.

### 2.4. Gene Expression Analysis Reveals a Poor Survival Subgroup of AcCC Tumors

**Figure 3:**
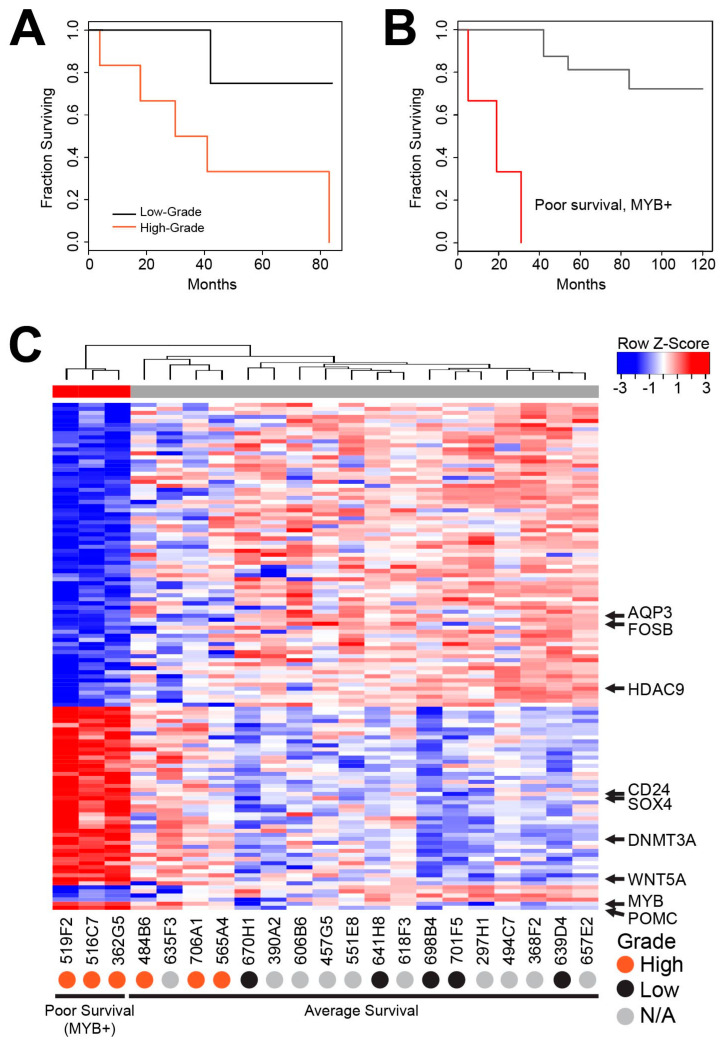
Poor survival subgroup of AcCC Tumors. (**A**) Kaplan–Meier survival curve comparing the high-grade transformation samples (orange) to low-grade samples (black). Note: grade information was not available for some samples, they were excluded; (**B**) Kaplan–Meier survival curves comparing the *MYB*-expressing (red) group from Figure 2 with all other samples (gray); (**C**) heatmap summarizes the differences in gene expression between the poor survival subgroup (left, red color bar) and the rest of the samples (gray color bar). Positions of some noteworthy genes are marked. Full-sized heatmap with all the genes labeled is provided in Figure S3.

We performed a more detailed gene expression analysis of the AcCC tumor samples including a survival analysis and found that one cluster (red and at lower left in the 3D plot in Figure 2A) showed much worse survival than the rest of the samples. High-grade transformation is an indicator of poor prognosis for AcCC tumors. Although we could not obtain grade information for our complete cohort, we did identify 6 high-grade and 5 low-grade samples. Figure 3A shows the comparison of their survival curves. The high-grade samples had a median survival of 36.5 months. In comparison, as shown in Figure 3B, the poor survival subgroup (red) that we identified by gene expression analysis is a subset of the high-grade samples and displayed a median survival of only 19 months, compared to 152 months for the rest of the cohort (gray). Thus, the poor survival of the high-grade samples appears to be largely due to the presence of a subset (the red samples) that has exceptionally poor survival.

We performed differential gene expression analysis, comparing just the poor survival (red) samples to the others and identified 1064 genes that were at least 2-fold up- or downregulated, with an adjusted *p*-Value less than or equal to 0.05. That analysis is summarized in the heatmap in Figure 3C, which shows the poor survival subgroup at the left (marked by red color bar at top), compared to all the other samples (gray color bar). A full-sized version of the heatmap, with all the genes labeled, is provided in Figure S3. Several interesting regulators were upregulated in the poor survival subgroup, including *MYB*, *WNT5A* and *SOX4* that encode transcription factors and *DNMT3A*, *CD24* and *POMC* that encode important regulators of cell differentiation and metabolism. Downregulated genes included *FOSB*, which encodes an important transcription factor, *HDAC9*, which encodes an epigenetic regulator and a gene that is strongly associated with salivary gland differentiation, *AQP3*, which encodes aquaporin 3 (Figure S3). Complete differential gene expression data are in Table S5. Additional Kaplan–Meier plots for the entire cohort and for males vs. females are provided in Figure S4.

### 2.5. AcCC Tumors with t(4;9) Overexpress NR4A3 Protein, which Interacts with Myb

**Figure 4:**
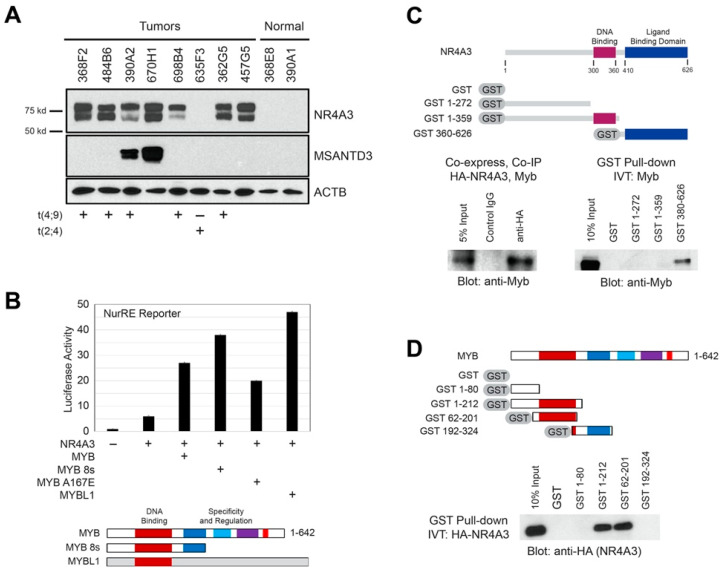
NR4A3 and MYB cooperate and interact. (**A**) Western blot analysis. Sample identifiers are shown at top, antibodies used to probe the blot are indicated at right. An actin loading control is shown at the bottom. The fusions that were detected (‘+’) or not detected (‘–’) by either RNA-seq or FISH are indicated along the bottom (blanks were not tested). High expression of NR4A3 protein was detected in all samples with t(4;9), but not in the normal samples (right) or the sample with t(2;4), which expressed the *NR4A2* gene instead. Two distinct NR4A3 proteins were detected; the main form (approximately 68kD, lower band) and an isoform (approximately 80kD, upper band). MSANTD3 protein was detected in the two samples that also had fusions activating that gene (390A2 and 670H1); (**B**) NR4A3 and Myb cooperate. HeLa cells were transfected with the NuRE promoter–luciferase vector plus plasmids expressing NR4A3 alone or with different Myb constructs, as indicated. The Myb proteins are diagrammed at the bottom. Additional controls are in Figure S5; (**C**) NR4A3 pull-down assays. The NR4A3 protein is diagrammed at top, including the DNA binding (red) and ligand binding (blue) domains. GST fusion proteins used for pull-down assays are diagrammed. Left panel: Cos-7 cells were co-transfected with plasmids expressing HA-tagged NR4A3 or Myb, protein complexes were immunoprecipitated using control IgG or anti-HA tag antibodies, and the complexes were detected by western blot using anti-Myb antibodies. Right panel: Myb protein was in vitro translated (IVT) and incubated with GST alone or GST-NR4A3 fusion proteins as indicated. The GST-bound complexes were isolated and assayed by western blot using anti-Myb antibodies; (**D**) Myb pull-down assays. The Myb protein is diagrammed, with several conserved domains shaded. GST fusion protein constructs are diagrammed. HA-tagged NR4A3 protein generated by in vitro translation was incubated with GST-Myb fusion proteins. The GST-bound complexes were isolated and assayed by western blot using anti-HA antibodies.

We evaluated the expression levels of the *NR4A3* and *MSANTD3* proteins in several AcCC samples, which were predicted to overexpress these proteins based on RNA-seq data. Figure 4A shows a Western blot analysis using antibodies specific for the NR4A3 or MSANTD3 proteins or the loading control, β-actin (ACTB). The results show two distinct bands for NR4A3 protein; the main form (approximately 68 kD, lower band) and an isoform (approximately 80 kD, upper band). Multiple isoforms of NR4A3 have been described previously [26] and alternative RNA splicing was evident in the RNA-seq data from the AcCC samples (not shown). The alternative RNA splicing is likely the source of the two isoforms of NR4A3 protein detected by western blotting. We detected NR4A3 protein overexpression in cases with t(4;9), but not in t(2;4) positive cases (e.g., 635F3), which is expected to express NR4A2 instead. As predicted by the RNA-seq data, we detected concurrent expression of NR4A3 and MSANTD3 proteins in tumors 390A2 and 670H1, which harbor fusion reads linked to both of these genes (Figure 1C, Table S2). Neither of the proteins were detected in normal salivary gland samples (right side of western blot). The combined results suggest that most, if not all AcCC tumors overexpress either the NR4A3 or NR4A2 genes and proteins, consistent with them being oncogenic drivers for these tumors.

NR4A3 and NR4A2 are members of a family of so-called nuclear orphan receptors: transcription factors related to steroid hormone receptors with DNA binding and transcriptional activation domains and are presumed to function by activating the transcription of other genes. We confirmed the transcriptional activity of NR4A3 using a mammalian one-hybrid assay (Figure S5). Briefly, cDNAs encoding full-length NR4A3 or several deletion mutants (diagrammed in Figure S5A) were subcloned into plasmid vectors encoding the DNA binding domain (DBD) of the yeast GAL4 protein. The plasmids expressing GAL4-NR4A3 fusions were co-transfected into mammalian cells along with a reporter gene construct containing a minimal promoter augmented with several binding sites for GAL4. Expression of the GAL4 DBD by itself did not activate the reporter, nor did GAL4 fused to the full-length NR4A3 (aa 1–626, Figure S5A). The N-terminal domain of NR4A3 showed autonomous transcriptional activation activity, as reported previously [27]. Neither of the constructs containing both the GAL4 and NR4A3 DNA binding domains were able to activate transcription in this assay, probably because the DNA binding domains interfere with each other in the one-hybrid system. Interestingly, the C-terminal ligand binding domain also activated transcription in both CV1 and HeLa cells. This indicated that NR4A3 transcriptional activity is mediated through both the N-terminal and C-terminal domains. Although we cannot exclude the possibility of endogenous ligands, the intrinsic activity of the ligand binding domain is likely mediated through protein–protein interactions with other cofactors.

### 2.6. Transcriptional Cooperativity between NR4A3 and Myb

Having observed overexpression of *NR4A3* and *MYB* genes in the poor survival subgroup, we determined whether NR4A3 and Myb proteins could functionally interact. We tested the transcriptional activity of NR4A3 using a reporter assay with a previously described [28] promoter containing the NurRE (Nur response element). When we transfected HeLa (or CV1, not shown) cells with the NurRE-luciferase reporter and a NR4A3 expression vector, we observed a dose-dependent increase in NurRE-reporter gene activity (Figure S5B), confirming that NR4A3 acts as a transcriptional activator [29].

Next, we tested whether expressed Myb protein could affect the transcriptional activity of NR4A3. As shown in Figure 4B, while NR4A3 activated the NurRE reporter by itself (6-fold), co-expression of full-length Myb led to much stronger activation (27-fold). Myb expression did not activate the NurRE reporter in the absence of NR4A3 (Figure S5C), suggesting that Myb did not bind and activate the promoter directly, but likely interacted with NR4A3 to enhance its activity in an indirect manner. In addition to full-length Myb, we tested an activated, oncogenic isoform of Myb lacking the C-terminal negative regulatory domain (MYB 8s), a version of Myb with a point mutation in the DNA binding domain that disrupts Myb DNA binding (MYB A167E) [30] and the related MYBL1 (A-Myb) transcription factor, which has a DNA binding domain that is nearly identical to the one in Myb, but little homology elsewhere. None of these activated the NurRE reporter on their own (Figure S5C), but all cooperated with NR4A3 to enhance activation of the NurRE reporter gene (Figure 4B), suggesting that Myb DNA binding is not required for the cooperation observed here. In addition, since only the DNA binding domains are conserved between MYB and MYBL1, the DNA binding domain of MYB is most likely to be responsible for interactions with NR4A3.

We also tested whether NR4A3 can enhance Myb protein activity. We utilized an *EN1*-promoter luciferase reporter gene, which contains Myb binding sites in the promoter, but lacks an NR4A3 response element [19]. As expected, Myb enhanced *EN1* gene reporter activity and the oncogenic Myb 8s was even more active. However, NR4A3 was not able to enhance the activity of Myb or Myb 8s. (Figure S5D) If anything, expression of NR4A3 led to slight repression of the EN1 gene promoter-reporter activity.

We tested whether there is a physical interaction between NR4A3 and Myb proteins. When NR4A3 and Myb were co-expressed in Cos7 cells, antibodies targeting the HA-tagged NR4A3 protein co-immunoprecipitated Myb, while control antibodies did not (Figure 4C, bottom left panel). We used GST pull-down assays to map the domains important for this interaction. In vitro transcribed/translated full-length Myb protein bound to GST-NR4A3 ligand binding domain (LBD, GST 360–626), but there was no interaction with GST only or with the N-terminal or DBD domains of NR4A3 (Figure 4C, bottom right panel. Full-sized blots are in Figure S8.). We also performed the reciprocal assay to identify the domains of Myb that interact with NR4A3. In vitro transcribed/translated full-length NR4A3 bound to the DNA binding domain of Myb (GST 1–212 and GST 62–201) (Figure 4D, bottom). Thus, Myb binds directly to the ligand binding domain of NR4A3, and NR4A3 binds to the DNA binding domain of Myb. The combined results suggest that the direct interactions between Myb and NR4A3 are responsible for the synergistic activation of the NurRE reporter gene shown in Figure 4B.

### 2.7. Cooperative Gene Activation by NR4A3 and Myb

Based on the synergy observed between NR4A3 and Myb protein activation of reporter genes, we investigated whether a similar cooperativity could be observed with regulation of endogenous genes. As AcCC cell lines are not available, we utilized NCI-H292 cells, a lung mucoepidermoid carcinoma cell line (MEC) that expresses very low levels of *NR4A3* and *MYB*, making it a suitable model to interrogate functional interactions between the NR4A3 and Myb proteins. The NCI-H292 cells were transduced with lentiviruses expressing NR4A3 or Myb alone or in combination. After 2 days, total RNA was harvested and RNA-seq analysis was performed.

**Figure 5:**
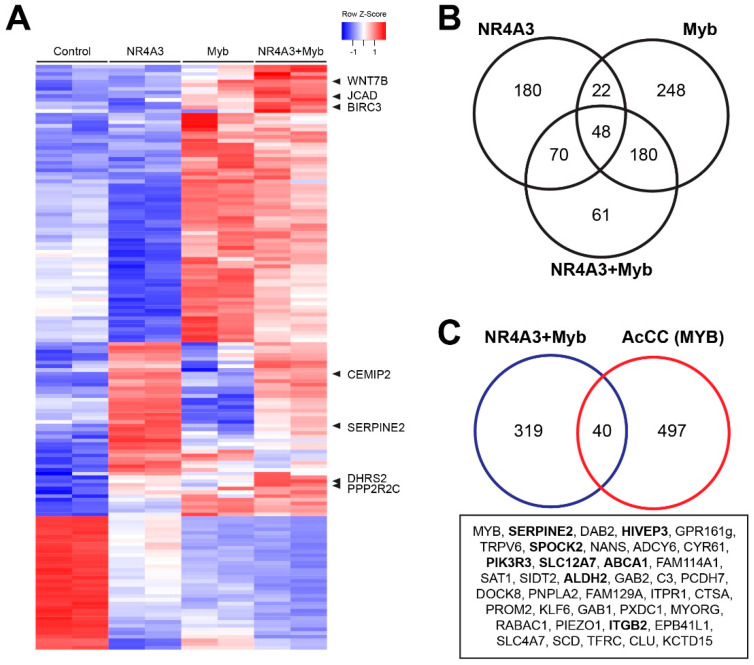
Differential gene expression induced by NR4A3 and Myb overexpression. (**A**) NCI-H292 mucoepidermoid cells were transduced with control (none), NR4A3, Myb or NR4A3 plus Myb. After 48 h, total RNA was isolated and RNA-seq analysis was performed. The heatmap summarizes the most significant differences in gene expression. Up- and downregulated genes are indicated by red and blue, respectively, as indicated in the key. Figure S6 has a larger version of this heatmap with all genes labeled; (**B**) Venn diagram comparing genes commonly regulated between NR4A3, Myb and NR4A3 plus Myb; (**C**) Venn diagram comparing genes commonly regulated by NR4A3 plus Myb vs. poor survivor subgroup of AcCC overexpressing *NR4A3* plus *MYB*. We identified 40 genes commonly regulated in both sets, which are listed in the box below the Venn diagram.

The results of the differential gene expression analysis among control (none), NR4A3, Myb or NR4A3 + Myb overexpression are summarized in the heatmap in Figure 5A. (Figure S6 has a full-sized heatmap with all the genes labeled.) Expression of NR4A3 led to upregulation (red) of a large set of genes, as did expression of Myb. We observed 320 genes differentially regulated by NR4A3 (more than 2-fold up- or downregulated compared to control with an adjusted *p*-Value less than 0.05), 498 genes regulated by Myb and 359 genes changed by expressing NR4A3 plus Myb. The Venn diagram in Figure 5B compares the genes affected by the three different treatments. Some of the most interesting genes are labeled in the heatmap in Figure 5A. For example, *SERPINE2* was strongly upregulated by NR4A3, but was not affected by ectopically expressed Myb. There were also genes that were much more strongly activated by the combination of NR4A3 plus Myb, including *WNT7B*, *JCAD*, *BIRC3*, *CEMIP2*, *DHRS2* and *PPP2RC* (labeled in Figure 5A).

We then compared the gene expression profiles of cells overexpressing NR4A3 plus Myb to poor survival AcCC tumor samples that also overexpress *NR4A3* and *MYB*. We identified 40 genes that were differentially regulated in both sets (Figure 5C). Among these genes, *SERPINE2* was expressed at high levels both in AcCC tumor samples and NCI-H292 cells after overexpression of both NR4A3 and Myb. Thus, we have identified a set of endogenous genes that are cooperatively regulated by NR4A3 and Myb, both in transduced NCI-H292 cells engineered to overexpress both proteins and in AcCC tumors that overexpress both genes. Some of the target genes may be critical downstream effectors in oncogenic actions of NR4A3 and Myb.

We verified some of the endogenous target genes that were identified in RNA-seq data by qPCR. We transduced NCI-H292 cells with empty vector, NR4A3, Myb or NR4A3 plus Myb combination and performed qPCR analysis. The results indicated at least three patterns of gene regulation: ENO3 and SERPINE2 expression were strongly induced by NR4A3 overexpression, but Myb did not significantly enhance their expression compared to NR4A3 alone (Figure S7). ACTA1 and CEMIP2 expression were induced by NR4A3 and adding Myb further enhanced this effect (Figure S7), consistent with the coactivator activity of Myb observed in transient transfection assay (Figure 4B). Interestingly, JCAD expression was induced by Myb, but not by NR4A3 (Figure S7). However, the combination of both strongly enhanced JCAD expression, indicating that NR4A3 and Myb showed transcriptional cooperation with another mechanism.

### 2.8. NR4A3 and Myb Cooperate in Colony Formation Assays

**Figure 6:**
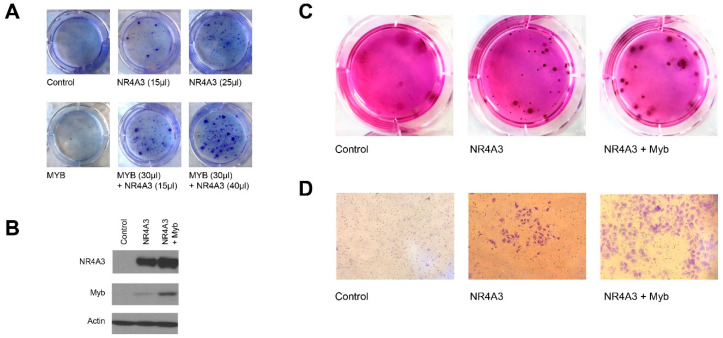
NR4A3 and MYB overexpression induce transformation of RK3E epithelial cells. (**A**) Rat kidney epithelial cells (RK3E) immortalized with E1A adenovirus were transduced with lentivirus containing empty vector (EV), NR4A3, Myb or the combination as indicated. For the NR4A3 plus Myb combination, RK3E cells were initially transduced with NR4A3 for 3 weeks. Then, Myb virus was added for 10 days. At day 31, cells were fixed, and images were captured; (**B**) NR4A3 and Myb protein expression. Transformed clones were isolated, and cell extracts were subjected to immunoblot with the indicated antibodies; (**C**) anchorage-independent growth assay. Transformed colonies from RK3E assay were isolated and seeded at 100–300 cells per well in media containing methylcellulose and grown for 11 days; (**D**) Boyden chamber assay. Transformed colonies were seeded in the 6 well Matrigel invasion chamber with 8-micron pores. Invasive cells were fixed and stained after 22 h.

We tested the transforming ability of NR4A3, Myb or NR4A3 plus Myb in rat kidney epithelial cells immortalized with adenovirus E1A (RK3E). This focus-forming assay was previously used to show oncogenic activity of RAS and Mect1–Maml2 oncogenes [31]. After lentiviral transduction to express NR4A3, we observed formation of 2–7 foci after 4 weeks (Figure 6A). The control group (none or empty vector) and Myb alone did not induce any foci. The combination of NR4A3 plus Myb induced the formation of 10–15 transformed foci. We observed that the combination of NR4A3 and Myb led to a greater number of foci and greater size of foci compared to NR4A3 alone (Figure 6A).

We isolated some of the foci formed in these assays and verified that the NR4A3 and Myb proteins were expressed (Figure 6B), then tested them in anchorage-independent growth and invasion assays. We observed a dramatic increase in the ability of NR4A3 and Myb transduced cells to form anchorage-independent colonies compared to control, which did not show any anchorage independent growth (Figure 6C). Interestingly, cells transduced with NR4A3 alone displayed some anchorage independent growth, but the cells grew at a much slower rate and formed smaller spheres compared to NR4A3 plus Myb expressing clones. These findings suggest that NR4A3 overexpression leads to anchorage-independent growth and the addition of Myb overexpression accelerates the growth of the NR4A3 transformed cells.

We used a Boyden chamber assay to test the invasiveness of the transformed cells. We observed that the NR4A3 plus Myb overexpressing cells showed highly invasive potential (Figure 6D) compared to control cells. The NR4A3 overexpression alone also led to increased invasiveness compared to control cells. These data support the notion of oncogenic cooperation between NR4A3 and Myb by their ability to enhance anchorage independent growth and invasive ability.

## 3. Discussion

We report that most, if not all salivary gland acinic cell carcinomas (AcCC) harbor translocations that result in activation of the *NR4A3* or *NR4A2* genes. The translocations do not lead to fusion transcripts or novel fusion proteins, but transpose transcriptionally active enhancer regions of chromosomes 4, 8 or 12 to the promoter regions of the *NR4A3* or *NR4A2* genes on chromosomes 9 and 2, respectively, leading to their upregulation. As reported by others [9] and confirmed here, the translocations appear to juxtapose a tissue-specific enhancer region to the *NR4A3* or *NR4A2* genes, which become overexpressed and likely function as oncogenic driver genes.

NR4A3 and NR4A2 are members of the NR4A or Nur orphan receptors subfamily and function as transcription factors in response to a wide range of stimuli [32,33]. Critical cellular pathways, including the NFKappaB [34] and Wnt signaling [35,36] have been reported to be the targets of the NR4A family member activation. Intriguingly, NR4A1, a member of the NR4A subfamily and closely related to NR4A3, regulates the R15 gene encoding the proline rich protein in salivary acinar cells of rodents [37]. Although all NR4A family members are widely expressed in different tissue types, they are not detected in normal parotid glands lending further support to the association between their activation and AcCC tumorigenesis.

Our data indicate that *NR4A3* and *NR4A2* are overexpressed, oncogenic drivers in the development of AcCC [32,38]. The activities of NR4A3 protein are likely cell type- or tissue-specific since the gene has been described as both an oncogene and as a tumor suppressor in different tumor types. *NR4A3* expression has also been linked to oncogenesis in extraskeletal myxoid chondrosarcoma [17,39] and its overexpression in pancreatic β-cell proliferation [40,41] and liver regeneration [42]. This contrasts to reports that *NR4A3* acts as a tumor suppressor gene in lymphoreticular [43] and follicular thyroid cancers [44]. Evidence that *NR4A3* expression enhances the apoptotic response of lymphoma and acute myeloid leukemia to chemotherapy support its classification as a tumor suppressor in these malignancies [45,46]. The differences in activities are probably due to tissue-specific regulation of different sets of target genes in different tumor types, suggesting that NR4A3 protein likely interacts with tissue-specific cofactors that help regulate its specificity and activity. The identification of those factors or mechanisms leading to the tissue-specific regulation of NR4A3 protein activity could be important for explaining how AcCC and other *NR4A3* expressing tumors develop.

Here, we have identified the oncogenic transcription factor Myb as one such cofactor. Gene expression analysis of a poor survival subgroup of AcCC tumors indicated overexpression of both *NR4A3* and *MYB*. We provide evidence that Myb and NR4A3 proteins directly interact: the ligand binding domain of NR4A3 interacts with the DNA binding domain of Myb, and that association increases the transcriptional activation activity of NR4A3. We have also identified endogenous target genes cooperatively regulated in NCI-H292 cells. Based on the differential gene expression data, Myb likely can also cooperate with NR4A3 when transcription factor binding sites are present for both proteins.

As nuclear orphan receptors, both NR4A3 and NR4A2 proteins contain conserved ligand binding domains in their C-terminal regions. Our studies agree with published results that NR4A3 contains transcriptional activation domains and likely acts to upregulate the expression of target genes [27]. However, it is possible that ligands or drugs could be identified that could be used to regulate the activity of NR4A3 protein in tumors. If ligands were identified it may be possible to design a therapeutic strategy to inhibit the activity of the overexpressed NR4Ax proteins. Several types of compounds have been reported to bind to and potentially regulate the ligand binding domain of NR4A3 [27,47]. However, there are not yet any studies describing whether these or other compounds may be able to mitigate the transcriptional or oncogenic activities of NR4A3 or NR4A2 in tumor cells. Alternatively, since the ligand binding domain of NR4A3 interacts with Myb, small molecule inhibitors against this interaction could be developed. This approach could be particularly useful since poor survival groups will likely benefit from additional targeted therapy.

Our transcriptomic profiling of AcCC tumors led to the identification of potential chromosomal translocations, which we validated using FISH. The fusion reads that we detected crossing chromosomal breakpoints were the result of ‘read-through’ transcription from a highly transcriptionally active region into the promoter region of the *NR4A3* or *NR4A2* genes. However, unlike fusion transcripts that occur in other salivary gland tumors, which result in the production of fusion proteins like the *MYB-NFIB* fusion genes produced in adenoid cystic carcinoma [19,21], in this case the result is a more highly transcribed *NR4A3* or *NR4A2* gene, but no fusion proteins. The RNA-seq analysis also allowed us to compare the gene expression phenotypes of our cohort of AcCC tumors and led to the identification of several subgroups. One subgroup identified by hierarchical clustering had a much worse survival than the others and also had a unique gene expression profile, with more than 700 significantly differentially expressed genes compared to the other AcCC tumors. This suggests that a gene panel biomarker could be developed to identify a subset of very high risk AcCC patients, who could then be stratified for more intensive follow-up or enrolled in clinical trials for personalized or targeted therapies. In the era of personalized and precision medicine, a major goal of translational studies is the development of biomarkers to identify special subgroups of patients with differentially activated pathways and potentially unique targetable driver genes. Confirmation of our results will require validation with larger, independent cohorts of patient samples, but could lead to important improvements for AcCC patients.

## 4. Materials and Methods

### 4.1. Tissue Collection

We performed in-depth RNA sequencing (RNA-seq) on 23 fresh tumor samples of primary salivary gland acinic cell carcinomas (AcCC) from equal number of patients treated primarily at MD Anderson Cancer Center. Only 21 samples yielded high-quality results that were used for the studies reported here. These samples were all collected between 1995 and 2018 at MD Anderson Cancer Center by specialized head and neck pathologists within 45 min from surgical excision. All samples were collected with informed consent of the donors, and studies were conducted in accordance with the principle of the Declaration of Helsinki. All studies were performed with Institutional Review Board-approved protocols. Two samples were excluded from our analyses due to quality control issues. Table S1 summarizes the clinical features of the samples.

### 4.2. RNA Isolation and Sequencing

Total RNA was extracted from fresh frozen tissues (30 mg) using RNeasy Universal Kit (Qiagen, 19300 Germantown Road, Germantown, MD, USA) according to the manufacturer’s instructions. Synthesis of cDNA and library preparation were performed using the SMARTer Universal Low Input RNA Kit for Sequencing (Takara Bio, 1290 Terra Bella Ave., Mountain View, CA, USA) and the Ion Plus fragment library kit (Thermo Fisher, 168 Third Avenue, Waltham, MA, USA) as previously described [19,20,21]. Sequencing was performed using the Ion Proton S5/XL systems (Thermo Fisher) in the Analytical and Translational Genomics Shared Resource at the University of New Mexico Comprehensive Cancer Center. RNA sequencing data are available for download from the NCBI BioProject database using study accession number PRJNA433667. Sequencing was performed using the Ion Proton S5/XL system, which has been shown to give superior results with poor-quality RNA samples [19,20,21]. The RNA-seq reads were aligned to the human genome (hg19) using both the standard TMAP aligner as well as with STAR, a ‘splice-aware’ aligner [48,49] that also detects candidate fusion reads mapped to more than one gene or chromosome.

### 4.3. Data Analysis

RNA-seq reads for each library were mapped to the human genome (hg19) using TMAP (v5.2.25, for gene expression) [50] and STAR (v2.5.3a, for translocation detection) [48,49]. Exon counts were calculated using htseq-count (v0.8.0, mode = union) against a RefSeq hg19 exons BED file with overlapping exons trimmed to remove duplicates and the TMAP aligned data, as described previously [19]. Exon counts for each gene were then summed to produce gene counts. Data were further analyzed for differential gene expression using the R packages edgeR, DESeq, plot3D, topGO and pathview [51,52], as described previously [19,20]. Translocations were identified either by STARfusion (v0.3.1) [48,49], using the STAR aligned data or by visual identification of chimeric reads using the integrative genomics viewer (IGV) (v2.3.79) [53], by comparing results from both aligners. High-quality human RNA-seq reads were aligned to the human genome (GRCh37; hg19) using TMAP (v5.0.7, Thermo Fisher, 168 Third Avenue, Waltham, MA, USA)) and gene counts were calculated using HT-Seq as previously described [19,21]. Poor quality samples, defined as those samples which had fewer than 10% of the median number of reads of all samples, were removed from further analyses. Chimeric reads were detected using the Integrative Genomics Viewer (version 2.3.79) [53,54] with “show soft clips” turned on, and then secondarily aligned to GRCh37/hg19 using BLAT at the UCSC genome browser [55,56] to determine the translocation partner. Pathway analysis was performed using Gene Set Enrichment Analysis [57].

### 4.4. Fluorescence In Situ Hybridization (FISH)

Tumor cells were fixed by touch preparations and FISH was performed to validate t(4;9) and t(2;4) translocation. Specific BAC clone probes for chromosome 4 targeted *CSN2, STATH*, *HTN3* and *HTN1* genes (RP11-529K3 and RP11-107G15, green), chromosome 9 targeted *NR4A3* (RP11-529K3, red), and chromosome 2 targeted *NR4A2* (RP11-605B16 and RP11-767C16, red, Empire Genomics, 1000 Youngs Rd Ste 207, Williamsville, NY, USA). Probes were hybridized according to the manufacturer’s protocols and signals from each probe were processed using the quantitative image processing system (Applied Imaging, 5555 Glenwood Hills Pkwy SE, Grand Rapids, MI, USA). In each case, 200 individual nuclei were scored and yellow signal was counted as positive when >3% of cells detected.

### 4.5. Protein Analysis by Western Blot

Tumor protein lysates were extracted using RIPA buffer containing protease inhibitor (no. 0505648900; Roche Applied Science, 200 Victoria Dr Gilroy, Gilroy, CA, USA) and phosphatase inhibitor (no. 04906837001; Roche Applied Science) cocktails. Laemmli SDS sample buffer (Bio-Rad, 2000 Alfred Nobel Drive, Hercules, CA, USA) was used for protein denaturation. Protein samples were loaded into SDS page gel and transferred onto nitrocellulose membranes. Membranes were incubated with primary antibodies; anti-NR4A3 (NGFI-B gamma/NOR-1, clone # H7833, mouse, R&D systems, 614 McKinley Place NE, Minneapolis, MN, USA), anti-MSANTD3 (#PA5-66513, rabbit, Thermo Fisher) and anti-ACTB (#AC-15, mouse; Sigma-Aldrich, PO Box 14508, St. Louis, MO, USA). After incubation with secondary antibody, bands were detected using SuperSignal West Pico PLUS substrate (# 34577, Thermo Fisher).

### 4.6. Plasmids

Mammalian expression vectors encoding Gal4-reporter, NurRE reporter, EN1 reporter, Myb, Myb 8s, Myb 8s-NFIB, A-Myb, Myb A167E were described previously [21,29]. NR4A3 expression vectors were cloned with HA epitope tags, encoded by pSG5.HA vectors containing SV40 and T7 promoters for expression in mammalian cells or in vitro transcription/translation. NR4A3 was also subcloned into pM vectors (Takara Bio, 1290 Terra Bella Ave., Mountain View, CA, USA) or pGEX4T1 vectors (GE Healthcare, 3000 N Grandview Blvd, Waukesha, WI, USA) for GST fusion protein expression.

### 4.7. Cell Culture and Transfections

Dulbecco’s modified Eagle’s medium (DMEM) with 10% fetal bovine serum was used to maintain CV-1, Hela, Cos-7, NCI-H292 and RK3E cells at 37 °C and 5% CO_2_.

For transient transfections, cells were seeded at 1 × 10^5^ cells/well in 12-well plates the day before transfection. Transfection of expression plasmids was performed using BioT transfection agent (Bioland Scientific LLC, 14925 Paramount Blvd Suite C, Paramount, CA, USA) according to the manufacturer’s protocol. After transfection, the cells were grown for 48 h. Cell lysis and luciferase assays on cell extracts were performed with Promega luciferase assay kit. The empty vector was used as controls for transfections of coactivator plasmids, and equal total DNA amounts were used in all samples in a given experiment. To control for variations in transfection efficiency, multiple independent transfection experiments were performed, and multiple plasmid preps for key plasmids were tested in these assays. The results are presented as the mean ± range of variation of two transfected wells from a single experiment, representative of at least three independent experiments.

### 4.8. Co-immunoprecipitation and GST-Pulldown Assays

For co-immunoprecipitation, Cos-7 cells were seeded 1 × 10^6^ cells/10-cm dish the day before transfection. Transfection of 6 µg of expression vectors was performed with Bio-T (Bioland) according to the manufacturer’s protocol. Cells were incubated for 48 h before whole cell extracts were made in 1 mL of radioimmune precipitation assay buffer (50-mM Tris HCl (pH 8.0), 150-mM NaCl, 2-mM EDTA, 1% Nonidet P-40, 0.5% sodium deoxycholate, 0.1% SDS). Co-immunoprecipitation and immunoblotting were performed as described previously [15]. The lysate was precleared by incubation with agarose protein A/G beads for 1 h and then immunoprecipitated overnight with 1–3 µg of the indicated antibodies and agarose protein A/G beads or magnetic beads. Immunoblotting was performed as described above.

For GST pull-down assays, glutathione S-transferase (GST) fusion proteins were produced in *Escherichia coli* strain BL21 and bound to glutathione agarose (Sigma-Aldrich) beads. NR4A3 or MYB fragments were cloned into the pGEX4T1 vector (GE Healthcare) for expression. NR4A3 or MYB was synthesized in vitro by transcription and translation using the TNT-T7 coupled reticulocyte lysate system (Promega, 2800 Woods Hollow Road, Madison, WI, USA). Each in vitro translation (10 uL) was incubated overnight at 4 °C with 1–2 micrograms of GST-fused protein bound to beads in NETN buffer [58]. Bound proteins were analyzed by immunoblot with anti-HA or anti-MYB antibody.

## 5. Conclusions

In summary, we provide evidence for recurrent chromosomal translocation of NR4A3 gene to highly active, tissue-specific enhancers present on three different chromosomes 4, 8 and 12. In addition, we also discovered one tumor sample with a highly homologous gene, NR4A2, chromosomal translocation to the enhancer region on chromosome 4, suggesting possible redundant functions of NR4A2 for NR4A3. These translocations result in aberrant overexpression of NR4A3 or NR4A2 genes and drive AcCC tumorigenesis. We also identified a poor survival subgroup that overexpress both NR4A3 and MYB. Importantly, there was a functional interaction between NR4A3 and MYB. MYB enhanced the transcriptional activity of NR4A3 as a co-activator. MYB binds to the ligand binding domain of NR4A3 while NR4A3 binds to the DNA binding domain of MYB. We identified endogenous target genes that are regulated by NR4A3 and MYB and provided evidence for transforming activity of NR4A3 and MYB, supporting their role as oncogenic drivers.

## Figures and Tables

**Table 1:** Correlation of fusion status and clinicopathologic features of acinic cell carcinomas (AcCC).

Parameter	Number (%)	Fusion		*p*-Value
		(+)	(−)	
**Age**				
35<	3 (14)	2	1	0.39
35≥	18 (86)	16	2	
**Sex**				
Male	4 (19)	3	1	1.00
Female	17 (81)	15	2	
**Tumor size**				
3 cm<	12 (57)	11	1	
3 cm≥	9 (43)	7	2	0.55
**Rec/met**				
yes	5 (24)	5	0	1.00
no	16 (76)	13	3	
**3-year survival**				
alive	17 (81)	15	2	1.00
dead	4 * (17)	3	1	

recurrence, met; metastasis. * two patients died with metastasis. (+) = positive; (−) = negative.

**Table 2:** New translocations and gene fusions of acinic cell carcinomas.

Translocation	Case Number (%)	Fusion Type	Number
t(4;9)	12 (57)	*HTN3-NR4A3*	3 *
		*STATH-NR4A3*	6
		*CSN2-NR4A3*	1
		*FDCSP-NR4A3*	1
		*CSN1S1-NR4A3* *HTN3-MSANTD3*	1 2 **
t(9:12)	4 (19)	*PRH1-NR4A3*	1
		*PRB3-NR4A3*	2 *
		*PRB1-STX17*	1
t(8;9)	1 (5)	*OXR1-NR4A3*	1
t(2;4)	1 (5)	*HTN3-NR4A2*	1
None	3 (14)		

* one case with *HTN3-NR4A3* and the other one case with *PRB3-NR4A3* have additional *HTN3-MSANTD3* fusion. ** both cases with *HTN3-MSANTD3* also have translocations to *NR4A3*, one from *HTN3* and one from *PRB3*.

## Supplementary Materials

The following are available online at https://www.mdpi.com/2072-6694/12/9/2433/s1, Figure S1: Clustering of translocation breakpoints in AcCC tumors on chromosome 4. Figure S2: Larger version of Figure 2C. Figure S3: Larger version of Figure 3C. Figure S4: Additional Kaplan–Meier survival curves. Figure S5: NurRE and EN1 promoter reporter assays. Figure S6: Larger version of Figure 5A. Figure S7: QPCR gene activation assays. Figure S8: Whole western blots from Figure 4. Table S1: Complete clinical information for acinic cell carcinoma samples. Table S2: Details of fusion reads, and translocations identified in AcCC samples. Table S3: Similar identification of t(4;9) translocations in previous reported samples. Table S4: Complete gene expression information from RNA-seq analysis of Acinic cell carcinoma. Table S5: Complete information about differentially expression genes comparing survival groups of acinic cell carcinoma.
