# Supplementary material for: Oncogenic Orphan Nuclear Receptor NR4A3 Interacts and Cooperates with MYB in Acinic Cell Carcinoma
Source: Cancers (Basel). 2020 Aug 27;12(9):2433. doi: 10.3390/cancers12092433 (PMC7565926; doi:10.3390/cancers12092433)
Supplement: Supplementary file 1 [file cancers-12-02433-s001.zip › cancers-892127-supplementary figures-final.pdf]

cancers

MDPI

Article

Oncogenic Orphan Nuclear Receptor NR4A3 Interacts and Cooperates with MYB in Acinic Cell Carcinoma

David Y. Lee, Kathryn J. Brayer, Yoshitsugu Mitani, Eric A. Burns, Pulivarthi H. Rao, Diana Bell, Michelle D. Williams, Renata Ferrarotto, Kristen B. Pytynia, Adel K. El-Naggar and Scott A. Ness

Supplementary Materials

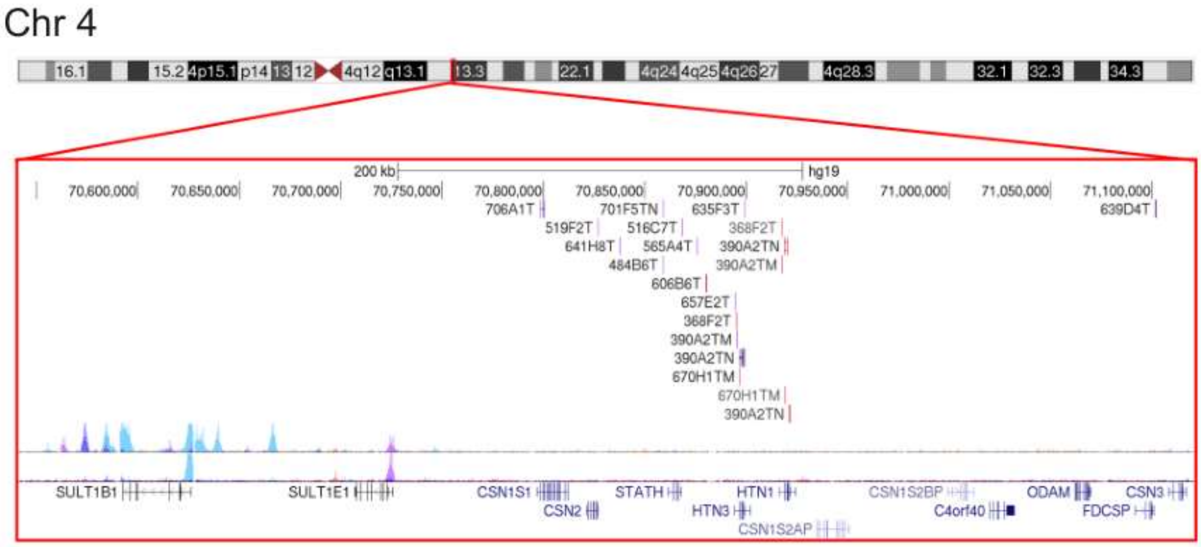

Figure S1. Clustering of Translocation Breakpoints in AcCC Tumors on Chromosome 4. RNA-seq fusion reads were used to map putative translocation breakpoints. Chromosome 4 breakpoints are shown. Diagram of the chromosome 4q13.3 region with locations of chromosome 4 translocations identified by RNA-seq fusion reads indicated by sample numbers (e.g. 706A1T). Note the clustering of breakpoints between the *CSN1S1* and *HTN1* genes. The locations of known genes are shown at bottom, with a UCSC Genome Browser track displaying known H3K27Ac (top) and H3K4Me3 (bottom) peaks, which were not determined in AcCC cells. A cluster of peaks indicating a possible super enhancer region is shown at left, near the *SULT1B1* gene.

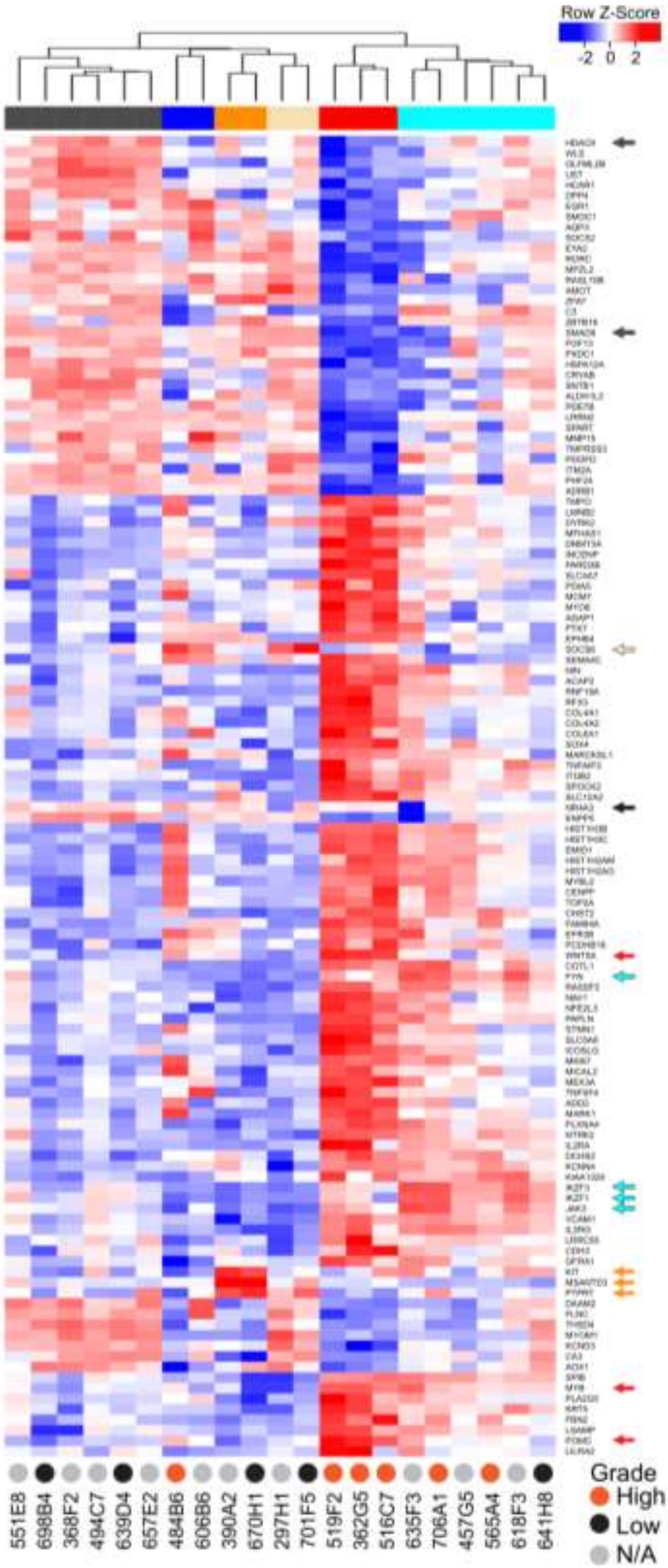

Figure S2. The heatmap summarizes the most significant differences in gene expression amongst the six subgroups of Acinic Cell Carcinoma samples analyzed by RNA-seq. The groups identified by hierarchical clustering are labeled at the top by the color bar beneath the dendrogram. The samples are labeled at the bottom, with the high-grade transformation samples marked with orange dots. The samples marked by black dots are not high-grade, and the samples without grade information are marked by gray dots. Genes are indicated at right, with interesting genes marked with arrows. (This is a larger version of Figure 2C).

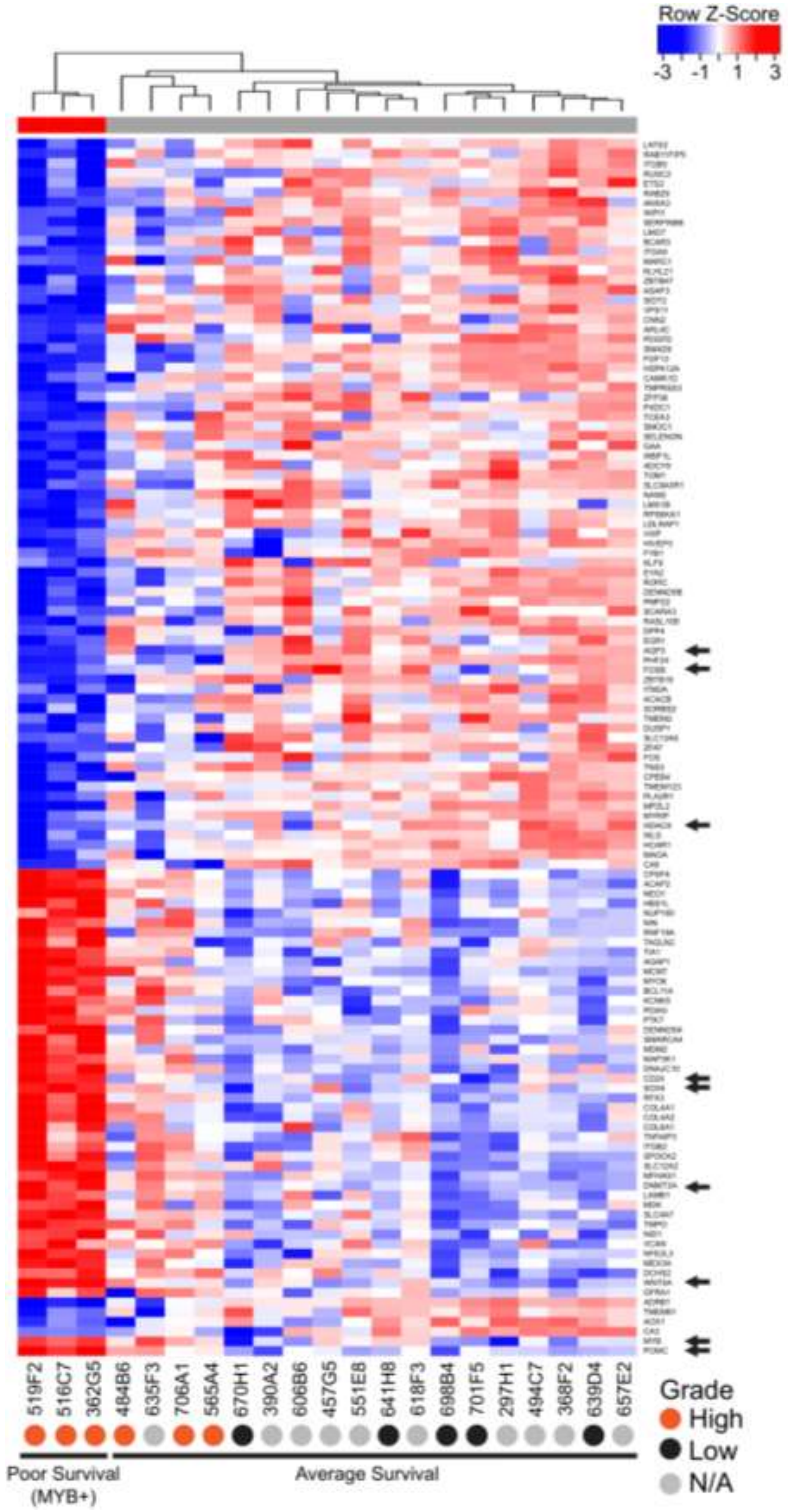

Figure S3. The heatmap summarizes the differences in gene expression between the poor survival subgroup (left, red color bar) and the rest of the samples (gray color bar). The positions of some noteworthy genes are marked. Grade information, where available, is shown at bottom (N/A = Not Available). (This is a larger version of Figure 3C).

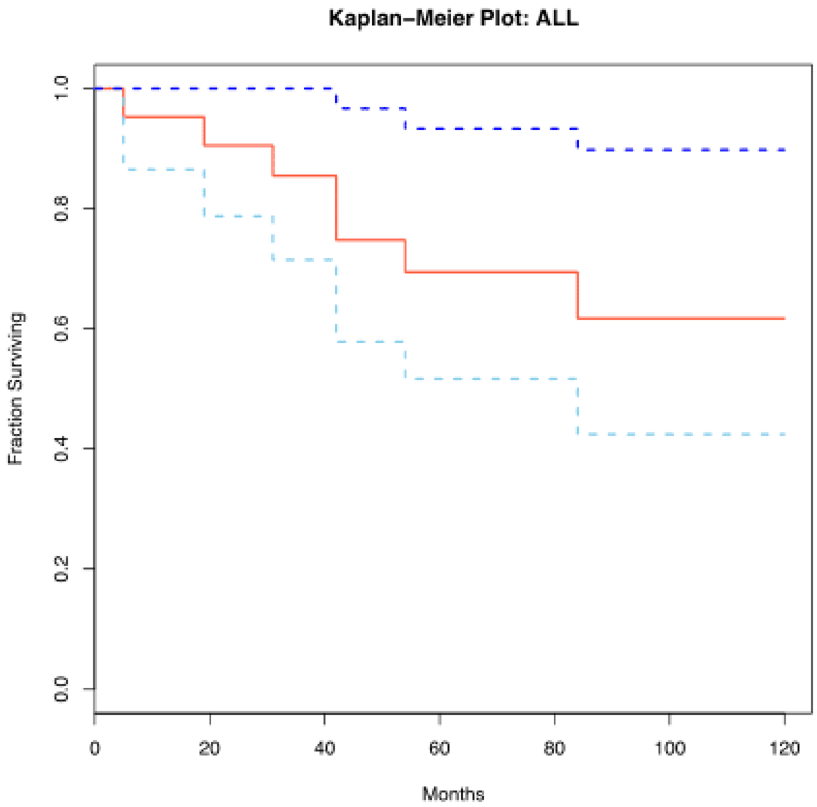

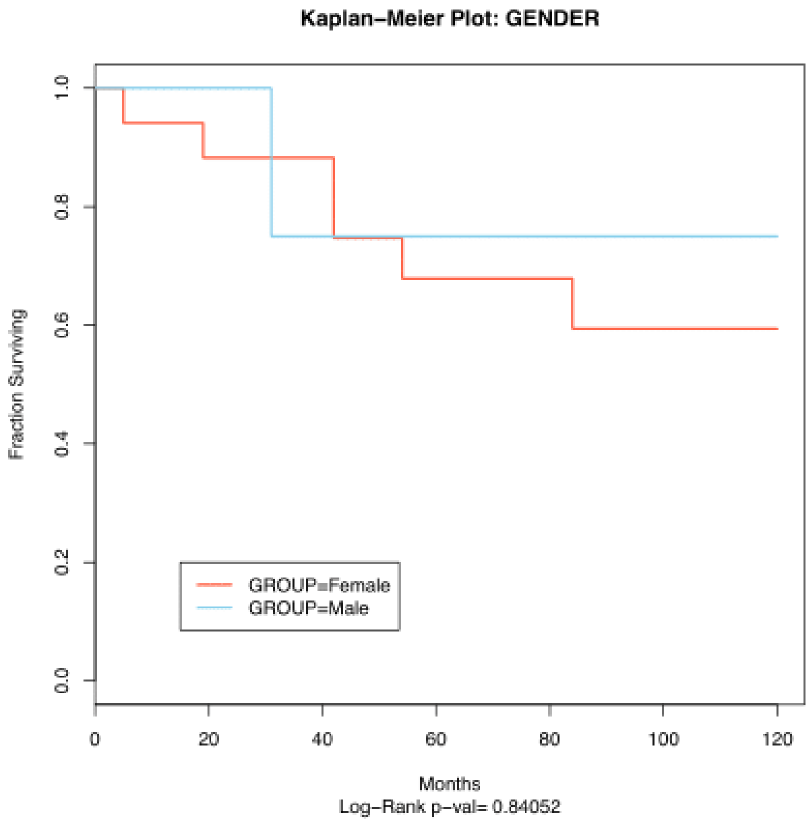

Figure S4. Additional Kaplan-Meier Plots. Kaplan-Meier survival plots are shown for all samples (top), with the upper and lower 95% confidence intervals shown by dotted lines. The entire cohort had a median survival of 142 months, with 91% survival at 23 months and 69% survival at 60 months. The lower plot compares the survival for Male and Female samples (not significantly different).

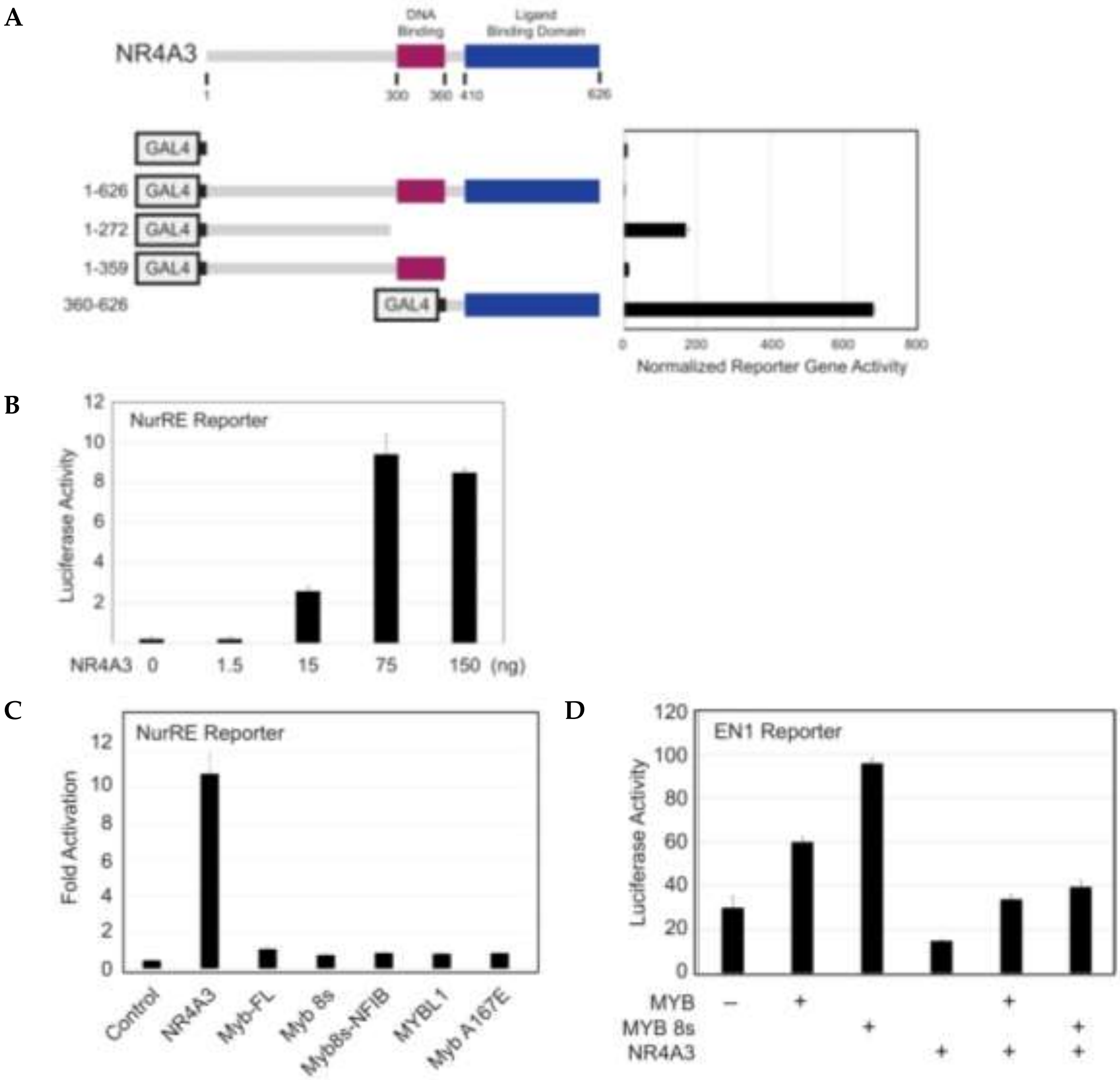

Figure S5. (**A**) One-hybrid transcriptional activation assays. At top is a diagram of NR4A3 protein structure showing conserved DNA binding (red) and ligand binding (blue) domains. **Left**: Diagrams of GAL4-NR4A3 fusion and deletion mutants. **Right**: Normalized reporter gene activity in transfected CV-1 cells. Note: Both the N-terminal domain and the ligand binding domain of NR4A3 displayed transcriptional activation activity in this assay. (**B**) NR4A3 activates a promoter containing the NurRE response element. HeLa cells were transfected with the NurRE promoter-luciferase vector [29]with increasing amounts of NR4A3 expression vector, as indicated along the bottom. Luciferase activity in arbitrary units is shown. Results shown are the mean + range of variation from 2 transfected wells from a single experiment, representative of at least three independent assays. (**C**) Activation of the NurRE Reporter. HeLa cells were transfected with NurRE reporter plasmid alone (Control) or with plasmids expressing NR4A3, full-length Myb (Myb-FL), Myb C-terminal deletion (Myb 8s), Myb fused to NFIB (Myb8s-NFIB), A-Myb (MYBL1) or Myb with a mutated DNA binding domain (Myb A167E). Only NR4A3 was able to activate the NurRE promoter. (**D**) Myb activation of the *EN1* promoter. CV-1 cells were transfected with an *EN1* promoter-luciferase reporter gene plus plasmids expressing full-length Myb, the Myb 8s deletion mutant (see Figure 3) and/or NR4A3, as indicated.

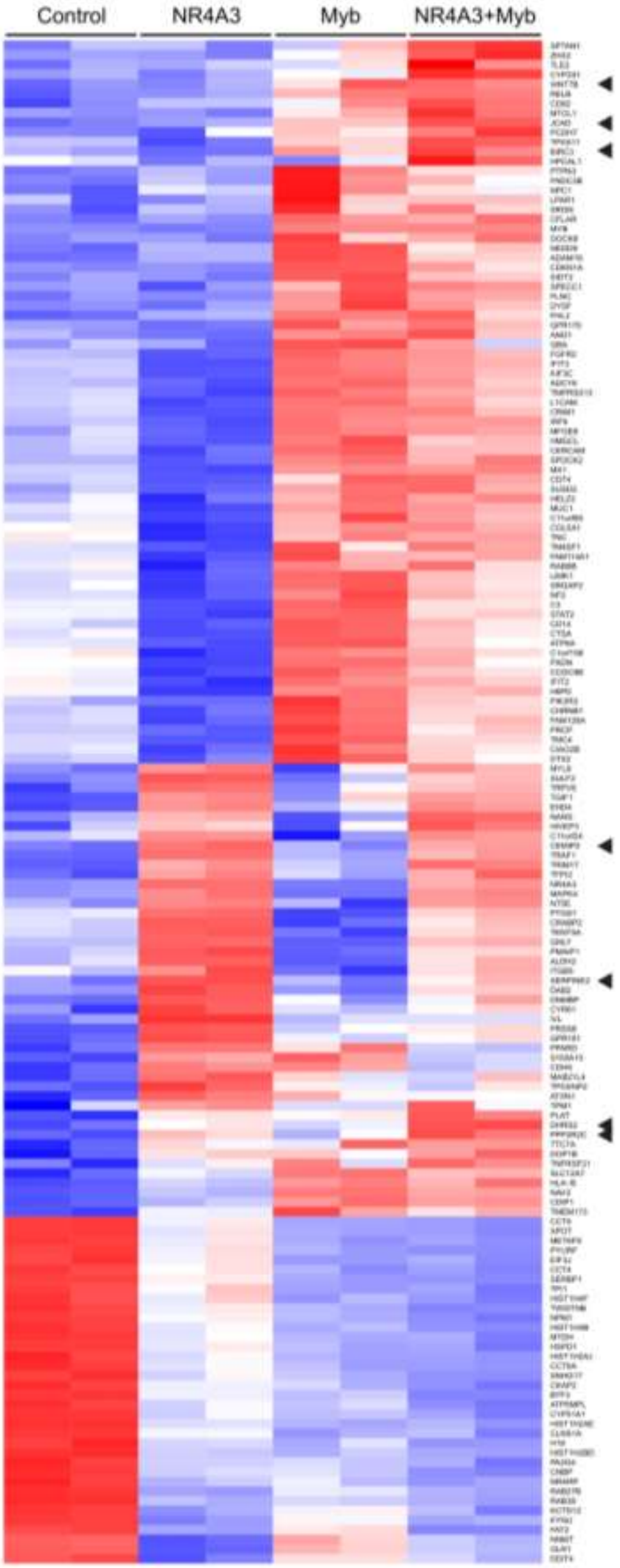

Figure S6. Cooperative Gene Activation by NR4A3 and Myb. This is a larger version of the heatmap shown in Figure 5A. See Figure 5 legend for details.

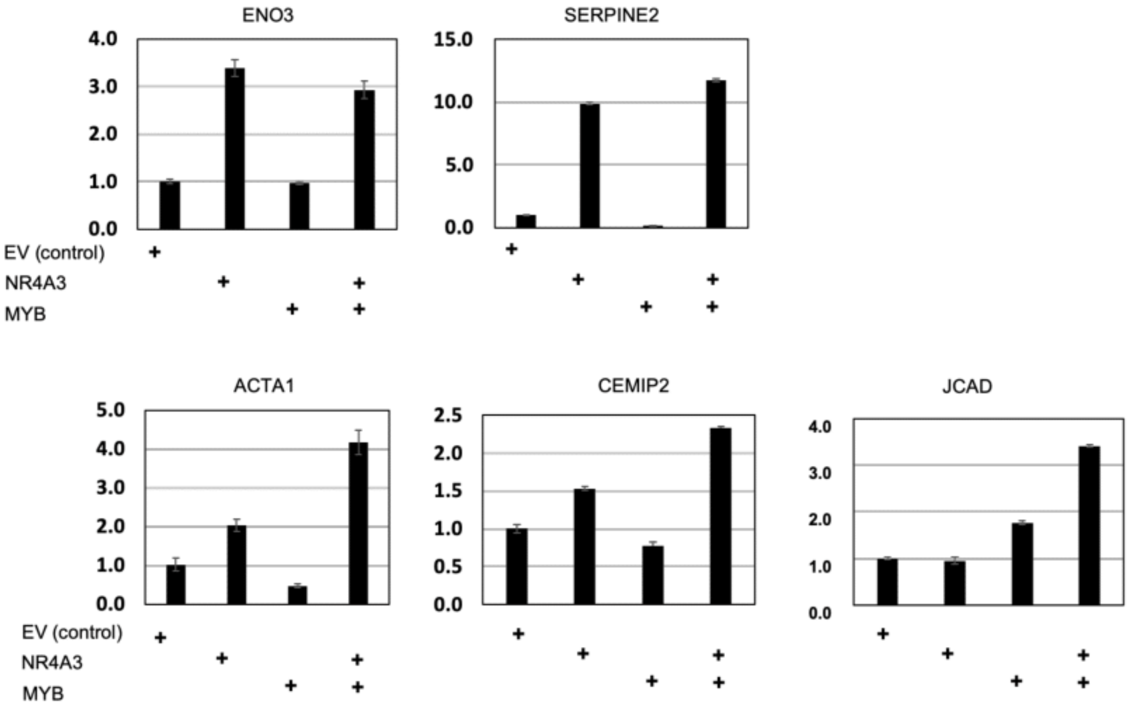

Figure S7. QPCR Gene Activation Assays. NCI-H292 cells were transfected with empty vector or plasmids expressing NR4A3, MYB, or NR4A3 plus MYB in combination. After 2 days, RNA was harvested and changes in gene expression were measured using QPCR. Genes assayed are listed above the plots, plasmids used are indicated below. See text for details.

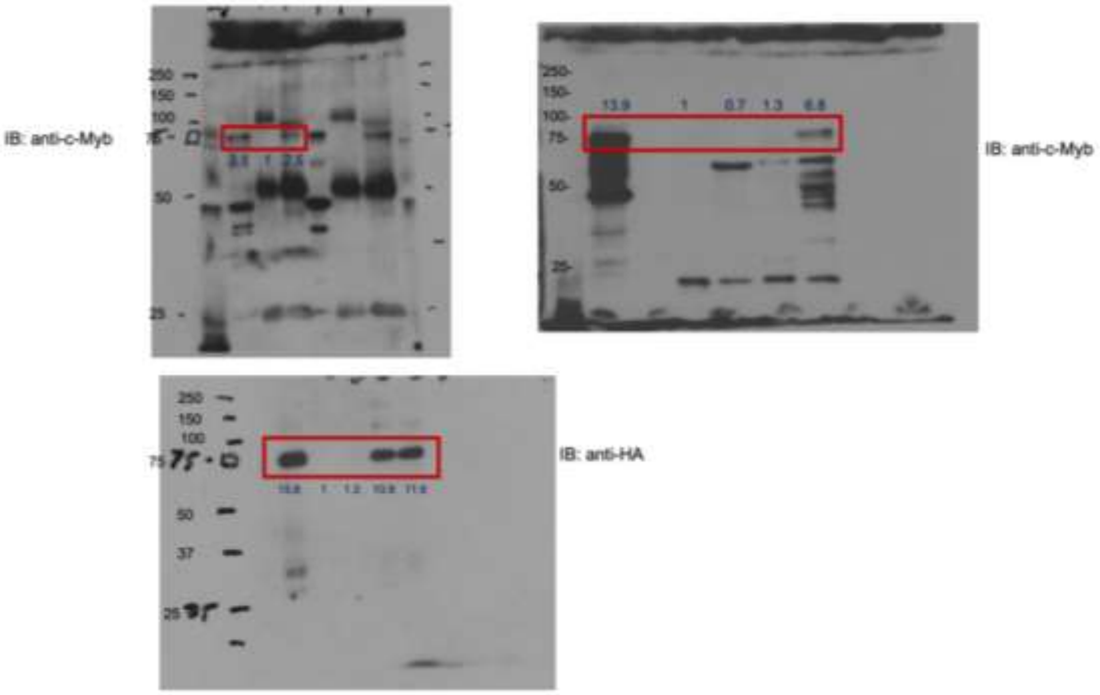

Figure S8. Whole Western Blots from Figure 4. These images show scans of the whole Western blots for the interaction assays in Figure 4. The sections shown in Figure 4 are boxed in red, and the numbers indicate the densitometry readings. Each blot also shows the molecular weight markers along the left side.
